# The E3 ubiquitin ligase RNF126 facilitates quality control of unimported mitochondrial membrane proteins

**DOI:** 10.1016/j.jbc.2025.108403

**Published:** 2025-03-12

**Authors:** Di Liu, Xin-Yu Huo, Xiaoli Zhang, Zai-Rong Zhang

**Affiliations:** 1 Interdisciplinary Research Center on Biology and Chemistry, Shanghai Institute of Organic Chemistry, Chinese Academy of Sciences, Shanghai, China; 2 Interdisciplinary Research Center on Biology and Chemistry, University of Chinese Academy of Sciences, Beijing, China

**Keywords:** mitochondrial membrane protein degradation, RNF126, ubiquilin, cytosolic quality control, ATP synthase F(0) complex subunit C1

## Abstract

Pathological stress can lead to failure in the translocation of mitochondrial proteins, resulting in accumulation of unimported proteins within the cytosol and upregulation of proteasome for their quality control. Malfunction or delay in protein clearance causes dysregulation of mitochondrial protein homeostasis, cellular toxicity, and diseases. Ubiquilins (UBQLNs) are known to serve as chaperone, which associates with unimported mitochondrial membrane protein precursors, and facilitates their proteasomal degradation. However, how UBQLN-engaged proteins are ubiquitinated and efficiently targeted to the proteasome are poorly understood. Here, using mitochondrial membrane protein ATP5G1 (ATP synthase F(0) complex subunit C1) as a model substrate, we report that E3 ubiquitin ligase RNF126 interacts with substrate-engaged UBQLN1, thereby promoting ubiquitination and degradation of unimported proteins during mitochondrial stress. We find that UBQLN1's ubiquitin-associated domain recruits RNF126 when its middle domain binds to unimported protein substrate. Recombinant RNF126 forms ternary complex with UBQLN1 and ATP5G1 precursor *in vitro* and catalyzes ubiquitination of UBQLN1-bound ATP5G1. Without RNF126, proteasomal degradation of ATP5G1 was compromised. These results explain how RNF126 and UBQLNs interplay to ensure specific quality control of unimported mitochondrial membrane proteins under pathophysiological conditions.

Mitochondria contain approximately 1000 to 1500 proteins, a large proportion of which bear transmembrane domains (TMDs). Mitochondrial membrane proteins are critical for maintaining the structural integrity and physiological functions of mitochondria (1). Most majority of mitochondrial proteins are encoded by the nucleus, synthesized in the cytoplasm, and transported to the mitochondria. Defective mitochondrial import leads to overaccumulation of mitochondrial protein precursors in the cytoplasm and increase of proteasome activity, suggesting enhanced protein degradation and quality control (2, 3). However, the concrete mechanism by which cytosolic mitochondrial membrane proteins are degraded in the cytoplasm remains unclear.

Dysfunction of mitochondria and alterations in protein homeostasis are associated with various human diseases (4, 5). Previous work has shown that in mouse models of early stage Huntington's disease, a mutant Htt protein (Httex1-97Q) blocks the mitochondrial protein import complex TIM23 channel, leading to defects in the translocation of mitochondrial precursor protein, mitochondrial dysfunction, and even neuronal death (6). In yeast, proteins including Ubx2, Cis1, and Msp1 facilitates releasing of entrapped precursor proteins from the mitochondrial outer membrane translocase, promoting their degradation *via* the proteasome (7, 8). In mammalian cells, E3 ligase complex UBR4–KCMF1–calmodulin can specially ubiquitinate nonimported proteins bearing mitochondrial targeting sequence (MTS) during import stress (9). However, it remains poorly understood how mitochondrial membrane proteins, when they fail to insert into mitochondria and accumulate in the cytosol, are ubiquitinated and eliminated by the quality control system.

Earlier work has shown that ubiquilin 1 (UBQLN1) interacts with unimported mitochondrial proteins containing TMDs with low or modest hydrophobicity (10). When mitochondrial membrane proteins fail to insert into mitochondria because of depolarization, UBQLN1 can recruit an E3 ligase to ubiquitinate substrates, thereby targeting the substrates for degradation by the proteasome (11). However, the identity of the specific E3 ligases involved in this process remains unclear.

In this study, we chose ATP synthase F(0) complex subunit C1 (ATP5G1), a mitochondrial inner membrane protein, as a model substrate to identify the key ubiquitin ligase (Fig. 1*A*). We discovered that unimported cytosolic ATP5G1 precursor (pATP5G1) undergoes ubiquitin-dependent proteasomal degradation and identified E3 ubiquitin ligase RNF126 for ATP5G1 ubiquitination. Furthermore, we purified recombinant UBQLN1–ATP5G1 complex and reconstituted ATP5G1 ubiquitination by RNF126. We proposed that RNF126 facilitates ubiquitination and the degradation of UBQLN1-bound mitochondrial membrane proteins.

**Figure 1:**
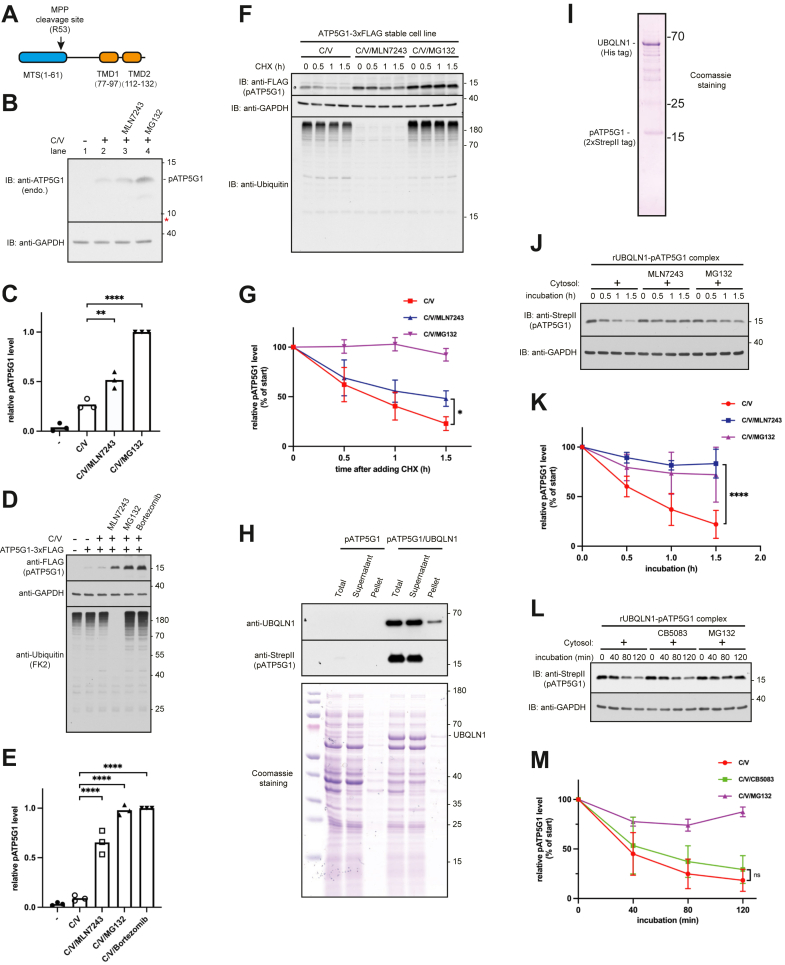
**Degradation of ATP5G1 precursor depends on ubiquitin–proteasome system**. *A*, diagram of human mitochondrial inner membrane protein ATP5G1, showing its predicted MPP protease cleavage site, N-terminal mitochondrial targeting sequence (MTS), and two TMDs. *B*, analyzing accumulation of endogenous pATP5G1 in HeLa cells treated with or without 4 μM CCCP, 2 μM valinomycin, 10 μM MLN7243, or 10 μM MG132 as indicated for 3 h. Total cell lysates were analyzed directly by immunoblotting for indicated proteins. The *red asterisk* indicates the proposed migration of the mature form ATP5G1, which was verified by independent studies. *C*, the quantification of the relative band intensities of pATP5G1 in (*B*) from three independent experiments was plotted and shown in the histogram. Individual data points represent independent biological replicates. ∗∗*p* ≤ 0.01, ∗∗∗∗*p* ≤ 0.0001 (Student's *t* test). *D*, cells expressing ATP5G1-3xFLAG were treated with or without 4 μM CCCP, 2 μM valinomycin, 10 μM MLN7243, 10 μM MG132, and 10 μM bortezomib as indicated for 3 h and analyzed by immunoblotting. *E*, the quantification of the relative band intensities of pATP5G1-3xFLAG in (*D*) from three independent experiments was plotted and shown in the histogram. Individual data points represent independent biological replicates. ∗∗∗∗*p* ≤ 0.0001(Student's *t* test). *F*, a cycloheximide (CHX) chase experiment to measure turnover rate of pATP5G1. Cells stably expressing ATP5G1-3xFLAG were pretreated with 4 μM CCCP and 2 μM valinomycin (C/V) for 45 min, followed by cotreatment with 10 μM MLN7243 or 10 μM MG132 for 15 min. Finally, CHX was added for time-course experiments. Total cell lysates were analyzed by immunoblotting for the indicated proteins. *G*, quantification of band intensities of pATP5G1 in (*F*). The errors represent the ±SD of three independent experiments. ∗*p* ≤ 0.05 (Student's *t* test). *H*, ATP5G1 was expressed without or with UBQLN1 in *Escherichia coli* BL21(DE3). The total lysates (T) were fractionated into a soluble supernatant (S) and pellet (P) and analyzed by immunoblotting and Coomassie blue staining. *Arrowheads* denote the UBQLN1. *I*, Coomassie staining of recombinant UBQLN1–pATP5G1 complex purified from *E. coli*. *J*, analyze pATP5G1 degradation in HEK293T cytosol. Purified rUBQLN1–pATP5G1 complex in (*I*) was added in untreated, 150 μM MLN7243-, or 150 μM MG132-treated cytosol fractions and incubated at 37 °C for indicated times. Reactions were stopped and directly analyzed by immunoblotting for pATP5G1. *K*, quantification of band intensities of pATP5G1 in (*J*). The errors represent the ±SD of three independent experiments. ∗∗∗∗*p* ≤ 0.0001 (Student's *t* test). *L*, analyze pATP5G1 degradation in HEK293T cytosol. Purified rUBQLN1–pATP5G1 complex in (*I*) was added in untreated, 150 μM CB5083-, or 150 μM MG132-treated cytosol fractions and incubated at 37 °C for indicated times. Reactions were stopped and directly analyzed by immunoblotting for pATP5G1. *M*, quantification of band intensities of pATP5G1 in (*L*). The errors represent the ±SD of three independent experiments. ATP5G1, ATP synthase F(0) complex subunit C1; CCCP, carbonyl cyanide m-chlorophenyl hydrazine; HEK293T, human embryonic kidney 293T cell line; MPP, mitochondrial processing peptidase; ns, not significant (Student's *t* test); pATP5G1, ATP5G1 precursor; TMD, transmembrane domain; UBQLN1, ubiquilin 1.

## Results

### Unimported ATP5G1 is degraded through the ubiquitin–proteasome pathway

We analyzed the fate of pATP5G1 failed in mitochondrial import. When cells were treated with CCCP (carbonyl cyanide *m*-chlorophenyl hydrazone) and valinomycin, compounds inducing mitochondrial depolarization, endogenous pATP5G1 bearing MTS accumulated in the cell and can be detected by antibody specific to ATP5G1's MTS (Fig. 1, *B*, lane 2, and *C*). Levels of pATP5G1 increased evidently (Fig. 1, *B* and *C*) when cells were coadministrated with MLN7243 or MG132, E1 or proteasome inhibitors, respectively, suggesting ubiquitin- and proteasome-dependent degradation. Similar results were observed when pATP5G1 was overexpressed in cells (Fig. 1, *D* and *E*), suggesting that plasmid-encoded ATP5G1 can be used to measure degradation in our experiments.

We further investigated substrate degradation by stopping protein synthesis with cycloheximide (CHX) in cells pretreated with CCCP and valinomycin together with either MLN7243 or MG132 (Fig. 1*F*). We observed an approximately 45 min half-life of pATP5G1 (Fig. 1, *F* and *G*). Importantly, treatment with MLN7243 or MG132 significantly slowed down pATP5G1 turnover, reducing the half-life to 1.5 h or more. Collectively, our results demonstrate that pATP5G1 accumulates during mitochondrial stress and is degraded through the ubiquitin–proteasome pathway.

Earlier work has reported that unimported mitochondrial membrane proteins bind to chaperone UBQLN1 in the cytosol (11). We then examined whether UBQLN1 could protect ATP5G1 from aqueous cytosol. To this end, full-length ATP5G1 was coexpressed with UBQLN1 or empty vector in bacteria *Escherichia coli*, a more simplified system compared with mammalian cell. We found that ATP5G1 was barely detected in bacteria without UBQLN1, suggesting little expression in the absence of UBQLN1 (Fig. 1*H*). When UBQLN1 was coexpressed, we were able to monitor ATP5G1, which existed exclusively in the supernatant, suggesting that ATP5G1's hydrophobic TMDs are solubilized in the aqueous solution, most likely by UBQLN1. We then successfully purified the recombinant UBQLN1–ATP5G1 complex (Fig. 1*I*), confirming that UBQLN1 can capture unimported pATP5G1 (11) and demonstrating that their interaction is direct.

To test whether UBQLN1-bound ATP5G1 can be degraded *in vitro*, we incubated the complex with the cytosolic fraction isolated from human embryonic kidney 293T (HEK293T) cells (Fig. 1*J*). We found that pATP5G1 in complex with UBQLN1 was rapidly degraded in the presence of the cytosol. Inhibiting either E1 or proteasome activity almost completely prevented substrate degradation (Fig. 1*K*), suggesting E1- and ubiquitination-dependent proteasomal degradation *in vitro*. Interestingly, ATP5G1 was still rapidly degraded in the presence of CB5083, a potent p97 ATPase inhibitor (Fig. 1, *L* and *M*), implying that degradation of unimported pATP5G1 does not depend on p97 ATPase activity. Taken together, these results indicate that unimported mitochondrial membrane proteins are primarily degraded by the ubiquitin–proteasome system. We are therefore interested in identifying the ubiquitin ligases involved in degrading UBQLN1-associated pATP5G1.

### RNF126 is necessary for the degradation of unimported pATP5G1

UBQLN1 and BAG6 are two common chaperones binding to membrane protein substrates (10, 12). Previous studies have suggested that BAG6 can recruit the ubiquitin ligase RNF126 for substrate ubiquitination (13, 14), and RNF126 can directly ubiquitinate the unimported soluble mitochondrial protein frataxin (15). We therefore investigated whether RNF126 might play roles in the ubiquitination and degradation of mitochondrial membrane proteins shielded by UBQLN1.

**Figure 2:**
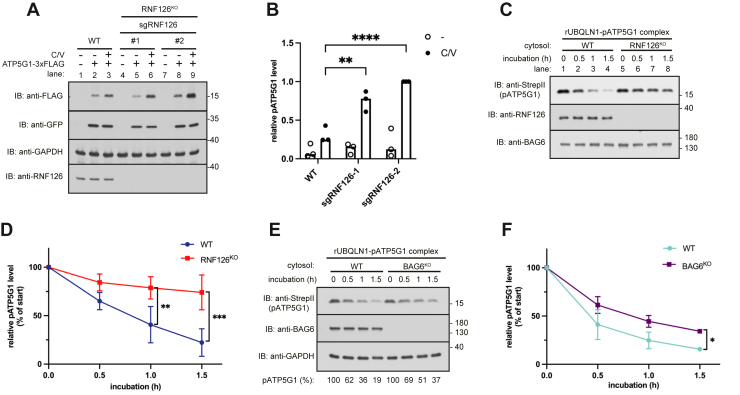
**RNF126 is necessary for the degradation of pATP5G1**. *A*, analyze level of ATP5G1-3xFLAG in WT and two RNF126 knockout cells (generated by sgRNF126-1 and sgRNF126-2) treated with or without 4 μM CCCP and 2 μM valinomycin. Total lysates were analyzed by immunoblotting. GAPDH and GFP were served as loading and transfection controls, respectively. *B*, the quantification of the relative band intensities of pATP5G1 in (*A*) from three independent experiments was plotted and shown in the histogram. Individual data points represent independent biological replicates. Statistical significance was determined using Student's *t* test. ∗∗*p* ≤ 0.01, ∗∗∗∗*p* ≤ 0.0001. *C*, analyze pATP5G1 degradation in WT and RNF126 knockout cytosolic fractions. Purified rUBQLN1–pATP5G1 complex (Fig. 1*I*) was added into indicated fractions and incubated at 37 °C for indicated times. Reactions were stopped and directly analyzed by immunoblotting for pATP5G1. *D*, quantification of band intensities of pATP5G1 in (*C*). The errors represent the ±SD of three independent experiments. ∗∗*p* ≤ 0.01, ∗∗∗*p* ≤ 0.001 (Student's *t* test). *E*, analyze ATP5G1 degradation in WT and BAG6 knockout cytosolic fractions. Purified rUBQLN1–ATP5G1 complex (Fig. 1*I*) was added into indicated fractions and incubated at 37 °C for indicated times. Reactions were stopped and directly analyzed by immunoblotting for pATP5G1. *F*, quantification of band intensities of pATP5G1 in (*E*). The errors represent the ±SD of three independent experiments. ∗*p* ≤ 0.05 (Student's *t* test). ATP5G1, ATP synthase F(0) complex subunit C1; CCCP, carbonyl cyanide *m*-chlorophenyl hydrazone; pATP5G1, ATP5G1 precursor; UBQLN1, ubiquilin 1.

To this end, we knocked out RNF126 by CRISPR–Cas9 technology and measure expression of pATP5G1 in cultured cells. The result showed that RNF126 depletion increased the level of pATP5G1 when mitochondrial import is impaired (Fig. 2*A*, lane 3 *versus* 6 and 9 and Fig. 2*B*) by CCCP and valinomycin, suggesting that RNF126 is required for proteasomal degradation of pATP5G1. We then investigated whether RNF126 promotes degradation of UBQLN1-bound pATP5G1. We isolated cytosolic fractions from wildtype and RNF126-depleted cells and incubated each with recombinant UBQLN1–pATP5G1 complex. The pATP5G1 showed a half-life of approximately 50 min when incubated with HEK293T cytosol containing endogenous RNF126 (Fig. 2, *C* and *D*), whereas the half-life extended to much longer than 1.5 h when RNF126 is absent (Fig. 2, *C* and *D*). Thus, we conclude that RNF126 promotes pATP5G1 turnover. Since chaperone BAG6 has been reported to bind to UBQLN1 (16), we then tested if BAG6 is required for the degradation of UBQLN1-bound pATP5G1. The recombinant complex was incubated with the cytosol from BAG6-depleted cells, and we observed a slightly impaired turnover of pATP5G1 (Fig. 2, *E* and *F*), suggesting that BAG6 modestly facilitates degradation of pATP5G1. Taken together, we propose that UBQLN1 may recruit RNF126 to ubiquitinate pATP5G1 and promote its degradation, with BAG6 having marginal impact on pATP5G1 turnover.

### UBQLN1 binds to TMD of pATP5G1 and recruits RNF126

**Figure 3:**
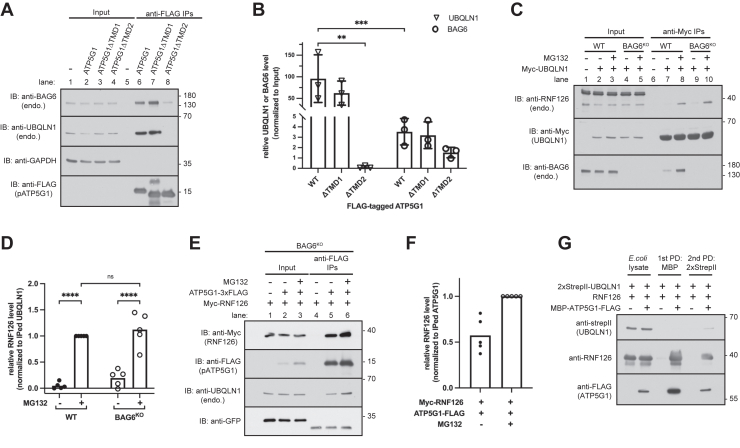
**UBQLN1 selectively binds to transmembrane domain (TMD) of ATP5G1 and interacts with pATP5G1 and RNF126 to form ternary complex**. *A*, ATP5G1 (WT) and deletion constructs lacking its TMD1 (ΔTMD1) or TMD2 (ΔTMD2) were transfected in cells. Total lysates were analyzed by immunoblotting either directly or after recovering ATP5G1 through anti-FLAG immunoprecipitations (IPs). *B*, Relative abundance of UBQLN1 or BAG6 was quantified by densitometric analysis of the blots in (*A*) and normalized to input levels in total lysates. Individual data points represent independent biological replicates. The errors represent the ±SD of three independent experiments. ∗∗*p* ≤ 0.01, ∗∗∗*p* ≤ 0.001 (two-way ANOVA). *C*, analyze the interaction of UBQLN1 with endogenous RNF126 in WT and BAG6 knockout cells. Cells expressing Myc-tagged UBQLN1 were treated with 20 μM MG132 (or DMSO as a control) for 2 h, and total lysates were then analyzed by immunoblotting directly or after anti-Myc IPs. *D*, relative abundance of RNF126 was quantified by densitometric analysis of the blots shown in (*C*) and normalized to anti-Myc immunoprecipitated UBQLN1. Individual data points represent independent biological replicates. ∗∗∗∗*p* ≤ 0.0001 (two-way ANOVA). *E*, analyze the interaction of ATP5G1 with RNF126 in BAG6 knockout cells. Cells expressing indicated proteins were treated with or without 20 μM MG132 for 2 h and analyzed by immunoblotting either directly or after recovering ATP5G1 through anti-FLAG IPs. *F*, relative abundance of RNF126 was quantified by densitometric analysis of the blots shown in (*E*) and normalized to ATP5G1 in anti-FLAG IPs. Individual data points represent independent biological replicates. *G*, two-step affinity purification of the UBQLN1–pATP5G1–RNF126 ternary complex. His-tagged RNF126 and 2xStrepII-UBQLN1 were coexpressed in *Escherichia coli* with or without MBP-ATP5G1-FLAG. Samples in indicated steps were analyzed by immunoblotting for ATP5G1, UBQLN1, and RNF126. ATP5G1, ATP synthase F(0) complex subunit C1; pATP5G1, ATP5G1 precursor; DMSO, dimethyl sulfoxide; MBP, maltose-binding protein; UBQLN1, ubiquilin 1.

To examine how pATP5G1 is recognized by UBQLN1, we deleted individual TMD of pATP5G1 (Fig. 3*A*) and analyzed interaction of these mutants with UBQLN1. Deleting TMD1 did not affect the interaction between pATP5G1 and UBQLN1, hinting that this region may not be critical for them to interact. However, the interaction between pATP5G1 and UBQLN1 was abolished upon deletion of TMD2, suggesting a critical role of TMD2 in binding to UBQLN1 (Fig. 3, *A* and *B*).

To test if UBQLN1 interacts with RNF126 in cells, we performed coimmunoprecipitation (co-IP) analysis. Since UBQLN1 binds to TMD-specific chaperone BAG6, which interacts with RNF126, we performed the experiment in both wildtype and BAG6 knockout cells (Fig. 3*C*). We did not observe interaction between UBQLN1 and RNF126 under normal, nonstressed condition. However, when proteasome activity was compromised with MG132, we noticed that UBQLN1 did coprecipitate with endogenous RNF126 (Fig. 3, *C*, lane 7 *versus* 8, and *D*). Importantly, the interaction remained when BAG6 was knocked out, strongly supporting BAG6-independent binding between UBQLN1 and RNF126. Because disruption of ubiquitin–proteasome pathway impeded degradation of unimported mitochondrial membrane protein substrates, these results suggest that UBQLN1 might associate with RNF126 only when substrate accumulated in the cell and are captured by UBQLN1 (11). Presumably, the UBQLN1–substrate complex could offer a new high-affinity surface for RNF126 binding. Therefore, we deduce that accumulation of substrates like pATP5G1 in cell might allow UBQLN1 to bind to RNF126, thereby facilitating substrate ubiquitination.

To further confirm this, we tested interaction between RNF126 and overexpressed ATP5G1, which accumulated as the pATP5G1 even without mitochondrial depolarization. We measured interaction by co-IP in the absence of the potentially confounding factor BAG6 because previous studies have shown that BAG6 recruits RNF126 for quality control of mislocalized or retrotranslocated membrane proteins (13, 14, 17). The result showed that pATP5G1 coprecipitated with RNF126 and UBQLN1 (Fig. 3, *E* and *F*), suggesting pATP5G1 might simultaneously bind UBQLN1 and RNF126. To further explore this possibility, we coexpressed maltose-binding protein (MBP)–tagged ATP5G1, 2xStrepII-tagged UBQLN1, and RNF126 in *E. coli*. The first purification for MBP showed that ATP5G1 binds to both UBQLN1 and RNF126. We then performed the second Strep-Tactin pulldown for UBQLN1 and observed the RNF126 in the elution fraction, implying that UBQLN1, pATP5G1, and RNF126 form ternary complex (Fig. 3*G*).

### Characterization of interaction between UBQLN1 and RNF126

**Figure 4:**
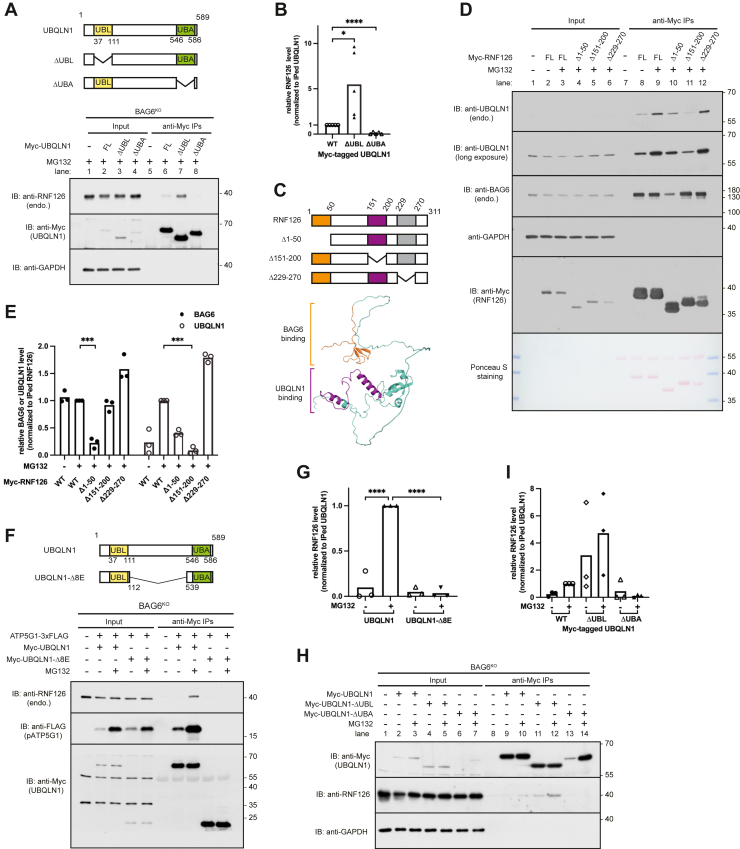
**Characterization of interaction between UBQLN1 and RNF126**. *A*, schematic representation of UBQLN1 (WT), a deletion construct lacking its UBL (ΔUBL) or UBA (ΔUBA) domain (*top*). Analysis of the interaction of UBQLN1 with endogenous RNF126 in BAG6 knockout cells (*bottom*). Cells expressing indicated proteins were treated with 20 μM MG132 for 2 h, and total lysates were analyzed by immunoblotting after recovering UBQLN1 through anti-Myc immunoprecipitations (IPs). *B*, relative abundance of RNF126 was quantified by densitometric analysis of the blots shown in (*A*) and normalized to anti-Myc immunoprecipitated UBQLN1. Individual data points represent independent biological replicates. ∗*p* ≤ 0.05, ∗∗∗∗*p* ≤ 0.0001 (Student's *t* test). *C*, schematic representation of RNF126 deletion mutants (*top*) and AlphaFold-predicted 3D structure of RNF126 (*bottom*). In the *bottom*: the *orange* domain (M1-G50) interacts with BAG6, whereas the *purple* domain (Q151-T200) interacts with UBQLN1. *D*, analyze the interaction of RNF126 mutants shown in (*C*) with UBQLN1. Cells were treated with or without 20 μM MG132 for 2 h and analyzed by immunoblotting and Ponceau S staining either directly or after recovering RNF126 through anti-Myc IPs. *E*, relative abundance of coprecipitated UBQLN1 or BAG6 was quantified by densitometric analysis of the immunoblots shown in (*D*) and normalized to RNF126 recovered by anti-Myc IPs. Individual data points represent independent biological replicates. ∗∗∗*p* ≤ 0.001 (Student's *t* test). *F*, schematic representation of UBQLN1 (WT) and UBQLN-Δ8E (exons 3–10 deletion construct) (*top*). In the *bottom*, BAG6 knockout cells expressing ATP5G1-3xFLAG and indicated constructs (*top*) were either untreated or treated with 20 μM MG132 for 2 h. Total lysates were analyzed by immunoblotting after recovering UBQLN1 using anti-Myc IPs. *G*, relative abundance of coprecipitated RNF126 was quantified by densitometric analysis of the immunoblots shown in (*F*) and normalized to UBQLN1 obtained by anti-Myc IPs. Individual data points represent independent biological replicates. ∗∗∗∗*p* ≤ 0.0001 (Student's *t* test). *H*, BAG6 knockout cells expressing UBQLN1 mutants were untreated or treated with 20 μM MG132 for 2 h and analyzed by immunoblotting after recovering UBQLN1 through anti-Myc IPs. *I*, relative abundance of coprecipitated RNF126 was quantified by densitometric analysis of the immunoblots shown in (*H*) and normalized to UBQLN1 enriched by anti-Myc IPs. Individual data points represent independent biological replicates. UBA, ubiquitin-associated domain; UBL, ubiquitin-like domain; UBQLN1, ubiquilin 1.

To determine how UBQLN1 recognizes RNF126, we deleted the ubiquitin-like (UBL) domain or ubiquitin-associated (UBA) domain of UBQLN1 and measured their interaction with RNF126 in the presence of MG132 (Fig. 4*A*). Co-IP experiment showed that deletion of the UBA domain in UBQLN1 entirely abolished its interaction with RNF126 (Fig. 4*A*, compares lane 6 and 8, and Fig. 4*B*). Strikingly, RNF126 interaction with the UBL-deleted mutant was enhanced (Fig. 4, *A*, compares lane 6 and 7, and *B*), consistent with earlier work showing that UBA domain is exposed when UBL domain is deleted or mutated (11). Thus, we conclude that the UBA domain of UBQLN1 is necessary for its interaction with RNF126.

To identify the specific region in RNF126 for interaction with UBQLN1, we made deletion mutants of RNF126 to test their interaction with UBQLN1 (Fig. 4, *C*–*E*). Co-IP experiment showed that when the sequence 151 to 200 was deleted, the amount of UBQLN1 associated with RNF126 evidently decreased, suggesting that this region is critical for binding to UBQLN1 (Fig. 4, *D*, lane 9 *versus* 11, and *E*). In contrast, we found that amino acid residues 1 to 50 of RNF126 is required for recruiting BAG6 (Fig. 4, *D* and *E*), consistent with earlier work (13, 18). This result suggests that the regions essential for the interaction between RNF126 and UBQLN1 does not overlap with those involved in the interaction between RNF126 and BAG6.

To identify if the presence of pATP5G1s is required for the interaction of UBQLN1 with RNF126, we performed co-IP analysis. We deleted UBQLN1's exons 3 to 10 (amino acids 113–538, 8 exons) to make UBQLN1-Δ8E mutant, which lacks the middle domain required for client binding (Fig. 4*F*, *top*) (11). While pATP5G1 and RNF126 markedly bound to wildtype UBQLN1, neither of them coprecipitated with UBQLN1-Δ8E (Fig. 4, *F*, *bottom* and *G*), suggesting that the middle region between UBL and UBA is required not only for client recruitment but also for RNF126 association. In addition, we noticed that UBQLN1 mutant lacking UBL domain interacted with RNF126 even in the absence of proteasome inhibitors (Fig. 4, *H*, lane 11 and *I*), consistent with the proteasome targeting function of the UBL. Thus, disruption of UBL might lead to accumulation of clients stalled on the middle region of UBQLN1, leading to enhanced RNF126 interaction (Fig. 4*H*). Thus, we conclude that the interaction between UBQLN1 and RNF126 is likely mediated by both UBA and cytosol-accumulated clients captured on the UBQLN1. When the UBL is deleted, the exposed “M” domain might provide an accessible interface for substrate or capture clients because of malfunction in the downstream degradation. Together with unbound UBA domain, which is required for E3 ligase recruitment, the middle region contributes to UBQLN1's interaction with RNF126.

### Recombinant RNF126 catalyzes ubiquitination of pATP5G1 and Omp25 *in vitro*

**Figure 5:**
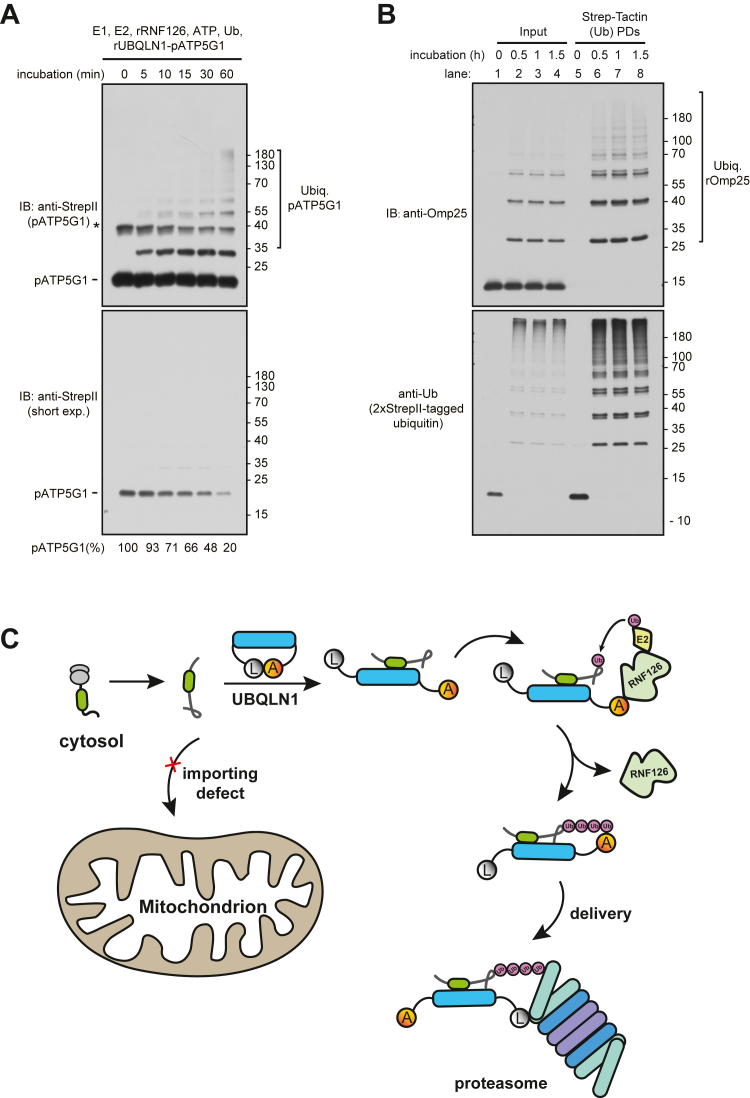
**RNF126 ubiquitinates UBQLN1's clients directly**. *A*, recombinant RNF126 (rRNF126) ubiquitinates StrepII-tagged pATP5G1 in complex with UBQLN1 *in vitro*. Purified UBQLN1–pATP5G1 (Fig. 1*I*) was incubated with recombinant Ube1, UbcH5b, His-tagged Ub, and RNF126 plus ATP at 25 °C for different times. The total reactions were directly analyzed by immunoblotting with anti-StrepII antibody. The *asterisk* represents residual ATP5G1 containing a noncleaved MBP fragment. *B*, purified UBQLN1–Omp25 complex was ubiquitinated as in (*A*), except with 2xStrepII-tagged ubiquitin. The total reactions were analyzed by immunoblotting either directly or after recovering ubiquitin through Strep-Tactin pull down. *C*, working model of UBQLN1–RNF126-mediated ubiquitination and degradation of unimported mitochondrial membrane proteins. MBP, maltose-binding protein; pATP5G1, ATP synthase F(0) complex subunit C1 precursor; UBQLN1, ubiquilin 1.

To test if RNF126 is sufficient to catalyze ubiquitination of pATP5G1 *in vitro*, we incubated recombinant RNF126 and UBQLN1–pATP5G1 complex, plus Ube1, UbcH5b, ATP, and ubiquitin. We observed monoubiquitination of pATP5G1 within 5 min of incubation, whereas polyubiquitination of pATP5G1 was observed within 30 min of reaction (Fig. 5*A*, *top*). In addition, about 20% of pATP5G1 left after 60 min reaction, indicating that majority of substrates are conjugated with ubiquitin (Fig. 5*A*, bottom). In addition, RNF126 ubiquitinated the recombinant Omp25 in complex with UBQLN1 (Fig. 5*B*). These results demonstrate that RNF126 functions as a ubiquitin ligase, catalyzing ubiquitination of pATP5G1 or Omp25 when they are bound to UBQLN1.

## Discussion

In this article, we have identified RNF126 as a ubiquitin ligase critical for the proteasomal degradation of mitochondrial membrane proteins that fail to import and become mislocalized in the cytosol under mitochondria stress. Based on its direct interaction with UBQLN1 and its demonstrated activity in ubiquitinating substrates, we propose that RNF126 accesses nonimported mitochondrial membrane proteins *via* UBQLN1. While RNF126 is recruited by UBQLN1 to ubiquitinate and facilitate degradation of pATP5G1 (Fig. 5*C*), RNF126 does not appear to target UBLQN1 when UBLQN1 is not engaged with substrates. Our findings provide a mechanistic explanation for earlier observations that depletion of RNF126 or UBQLN1 leads to the accumulation of mislocalized mitochondrial proteins in the cytosol, exacerbating mitochondrial stress and potentially causing cell death (19, 20).

The E3–chaperone pair RNF126–UBQLN1 plays unique roles in mitochondrial protein quality control. Recently, an E3 ligase complex containing UBR4, KCMF1, and calmodulin was shown to ubiquitinate MTSs in the cytosol during mitochondrial import stress (9). However, degradation pathway for unimported mitochondrial membrane proteins, particularly those lacking MTS, remains poorly understood. Our identification of RNF126–UBQLN1 complex provides insights into how unimported mitochondrial membrane proteins are selectively targeted for degradation. Earlier studies have shown that UBQLN1 can bind and promote the ubiquitination of mitochondrial membrane protein BCLb (21). RNF126 can ubiquitinate ferroptosis suppressor protein 1 (FSP1), which contains a weakly hydrophobic TMD, in the cytosol (22). We propose that UBQLN1 and RNF126 may cooperate to ubiquitinate membrane proteins with TMD of relatively mild hydrophobicity. In addition, RNF126 ubiquitinates and facilitates the degradation of mitochondrial matrix protein frataxin, although the mechanism by which frataxin is recognized by RNF126 remains unclear (15). It would be interesting to study whether frataxin contains a cryptic hydrophobic fragment that could be recognized by UBQLN1.

Impairment of proteasome activity results in the accumulation of no-degraded clients shielded by UBQLN1 under mitochondrial import stress. The UBQLN1-Δ8E mutant, which is unable to bind substrates, also fails to associate with RNF126 (Fig. 4*F*). This suggests that sustained substrate binding to UBQLN1 is required for RNF126 recruitment. Such substrate-dependent E3–chaperone association ensures that the ligase activity of RNF126 is tightly controlled and deployed only when substrates are engaged by UBQLN1.

Deletion of 151 to 200 domain in RNF126 almost completely abolished its interaction with UBQLN1 (Fig. 4, *D* and *E*), highlighting this domain as the core site for the interaction. In contrast, deletion of residues 1 to 50 reduced the interaction by approximately 50%, suggesting a stabilizing role in maintaining the proper overall conformation of RNF126. This conformation likely ensuring that the 151 to 200 region remains accessible for UBQLN1 binding. Together, these results indicate that both regions contribute to the stability and accessibility of the critical interaction site.

Previous results have shown that ubiquitin attached on the UBQLN1-bound clients interacts with UBQLN1 UBA domain, disrupting the UBL–UBA interaction and exposing UBL domain for proteasomal engagement (11, 23). Our results indicate that the UBA domain also recruits RNF126, which mediates substrate polyubiquitination. This polyubiquitin chain might interact more strongly with UBA domain than RNF126, facilitating RNF126 release from UBQLN1. With dual targeting signals, UBL and polyubiquitin, UBQLN1-bound substrates are efficiently delivered to the proteasome, enabling rapid degradation and maintaining quality control of unimported mitochondrial membrane proteins (Fig. 5*C*). Further investigations are needed to understand how ubiquitination, RNF126 release, and proteasome targeting are coordinated in this pathway.

As the most broadly expressed member of the UBQLN family, UBQLN1 has a large number of clients and regulates multiple cellular process, including endoplasmic reticulum–associated degradation and apoptosis (24, 25, 26, 27). Our identification of RNF126 as the obligate E3 ligase for UBQLN1-mediated client ubiquitination and degradation has broad implications for cellular physiology and pathology. RNF126 is upregulated in several cancer types and is essential for their proliferation (28). The upregulation of RNF126 in these cells coincides with an increase in mitochondrial biogenesis (29), suggesting that the RNF126-mediated quality control mechanism described here may contribute to the rapid propagation of these cancer cells. Moreover, depletion of UBQLN1 in mouse brain or B-cell lymphoma–derived cell also leads to increased oxidative stress and accumulation of mitochondrial proteins in the cytosol (20, 30, 31). Mitochondrial dysfunction and impaired import are also hallmarks of Alzheimer's disease (19, 32, 33), and mutations in the UBQLN1 “M” domain are considered risk factors for Alzheimer's disease (34). Enhancing UBQLN1–RNF126–mediated elimination of mislocalized mitochondrial membrane proteins may offer a potential therapeutic strategy for these diseases.

## Experimental procedures

### Plasmid, antibody, and other reagents

The DNA sequences encoding human RNF126, UBQLN1, Omp25, and ATP5G1 were amplified from RT–PCR products of total RNA extracted from HEK293T cells and cloned into the pCDNA5/FRT/TO vector with an N-terminal or C-terminal Myc, FLAG, or 3× FLAG tag, as indicated in the article. TMD1 and TMD2 deletion mutants of ATP5G1 were generated as described in Figure 1*A*. Mutants of UBQLN1 and RNF126 were made according to the diagrams described in Figure 4*A*, *C* and *F*, using Myc-UBQLN1 and Myc-RNF126 as templates.

For plasmids coexpressing UBQLN1 and pATP5G1 in bacteria, the coding regions of UBQLN1 (with an N-terminal 14xHis tag) and pATP5G1 (with an N-terminal MBP-3C tag and a C-terminal FLAG-2xStrepII tag) were inserted into multicloning site MCS-I and MCS-II, respectively, in the same pET-Duet vector. To make a single construct expressing UBQLN1 and Omp25, the coding regions of UBQLN1 (with an N-terminal 14xHis tag) and Omp25 (with an N-terminal MBP-3C-Myc tag) were inserted into MCS-I and MCS-II, respectively, in the same pET-Duet vector.

Antibodies were from the following sources: anti-UBQLN1 (Cell Signaling; catalog no.: 14526S), anti-ATP5G1 (Abcam; catalog no.: ab96655), anti-RNF126 (Abclonal; catalog no.: AB_2764884), anti-FLAG (E4195, in-house), M2 (Sigma–Aldrich), anti-Myc (E4193, in-house), FK2-HRP (Enzo; catalog no.: ENZ-ABS840HRP-0100), GAPDH-HRP (Proteintech; catalog no.: HRP-60004), anti-StrepII (Abclonal; catalog no.: AE066), and anti-Tubulin (Proteintech; catalog no.: 66031-1-Ig). Antibodies against BAG6 and GFP are previously described (35).

Small-molecule compounds are as follows: MG132 (MedChemExpress), CB5083 (MedChemExpress), CCCP (MedChemExpress), valinomycin (MedChemExpress), MLN7243 (MedChemExpress), and bortezomib (MedChemExpress), cOmplete protease inhibitor cocktail (Roche).

### Mammalian cell lines and transfections

All cells were maintained in Dulbecco's modified Eagle's medium (Thermo Fisher Scientific; catalog no.: C11995500BT) supplemented with 2 mM penicillin–streptomycin–glutamine (Thermo Fisher Scientific; catalog no.: 10378016) and 10% fetal bovine serum (GIBCO, Thermo Fisher Scientific; catalog no.: 10099-141). Transfections were performed using Lipofectamine 2000 (Thermo Fisher Scientific; catalog no.: 11668019) or PolyJet (SignaGen; catalog no.: SL100688) according to the manufacturer's instructions. We usually transfected around three-eighths of the recommended amount of DNA to achieve very low but detectable expression level, and GFP was cotransfected as a control to verify that the transfection efficiency was around 80% in all wells. Transfected cells were usually cultured for 24 to 36 h before further manipulation or analysis. Importantly, we generated the HEK293T Flp-In cell lines stably expressing ATP5G1-3xFLAG and characterized the substrate translocation and degradation. Transient transfection in normal cells produced similar results.

### Construction of knockout cell line using CRISPR–Cas9 technology

RNF126 knockout and BAG6 knockout cells were generated using CRISPR–Cas9 technology (36). Briefly, HEK293T cells were transfected with psPAX2 (Addgene; catalog no.: 12260), pMD2.G (Addgene; catalog no.: 12260), and pSpCas9(BB)-2A-puro (PX459) V2.0 plasmid (Addgene; catalog no.: 62988) containing sgRNF126-1 5′-CAGGCGCGGGACGATCTCCA, sgRNF126-2 5′-TCCGGGATGCGGCGACGCCT, or sgBAG6 5′-GAGGAGGAGCCCGTTCACC (14). Cells infected with sgRNA virus were selected by the addition of puromycin (2.5 μg/ml). Single cells were then plated into individual wells of 96-well plates. Knockout of RNF126 or BAG6 was validated by Western blotting.

### Native IPs or pulldowns

HEK293T cells expressing indicated proteins from 6 cm dishes were harvested with ice-cold 1x PBS, then lysed with 1 ml co-IP buffer (50 mM Tris–HCl, 150 mM NaCl, 2 mM MgCl_2_, and 0.05% digitonin [Sigma; catalog no.: D141] plus 1x protease inhibitor cocktail) on ice for 15 min. The cell lysates were subjected to centrifugation at 20,000*g* for 10 min to harvest cytosolic proteins (supernatant). The supernatants were incubated with 10 μl packed agarose with immobilized anti-FLAG or anti-Myc antibodies at 4 °C for 2 h. The agarose beads were washed three times with 1 ml co-IP buffer, eluted with 50 μl SDS sample buffer, and analyzed by immunoblotting.

### Protein expression and purification

Recombinant RNF126 was expressed by transforming the pDuet-ORF1 (6xHis-RNF126) plasmid into *E. coli* BL21(DE3) according to a previous report (14). Recombinant UBQLN1–pATP5G1 (rUBQLN1–pATP5G1) or rUBQLN1–Omp25 complex was obtained by expressing pDuet-ORF1 (MBP-3C-ATP5G1-FLAG-2xStrepII)-ORF2 (14xHis-UBQLN1) or pDuet-ORF1 (MBP-3C-Myc-Omp25)-ORF2 (14xHis-UBQLN1) in BL21(DE3), induced with 0.4 mM IPTG at 16 °C for 20 h. Cells were lysed with buffer A (50 mM Tris–HCl, pH 7.5, 200 mM NaCl, 5% glycerol, 1 mM β-mercaptoethanol, and 1 mM PMSF) using a French press at a pressure of 1000 bar. Cell debris were pelleted by centrifugation at 20,000*g* for 30 min. The cleared cell lysates were filtered through a 0.22 μm filter and then incubated with Dextrin beads (Smart-Lifesciences) for 2 h. After incubation, the beads were washed three times with buffer A. The target proteins were eluted by incubation with glutathione-*S*-transferase–tagged 3C protease at 4 °C for 2 h The elution fraction was collected and purified again with nickel–nitrilotriacetic acid beads (Smart-Lifesciences) for His-tagged UBQLN1, and subsequently, eluted with buffer A supplemented with 500 mM imidazole. The eluted proteins were evaluated by SDS-PAGE and Coomassie blue staining and stored at −80 °C.

To test the expression of pATP5G1, *E. coli* cells were cotransformed with either pDuet-ORF1 (MBP-3C-ATP5G1-FLAG-2xStrepII) or pDuet-ORF1(MBP-3C-ATP5G1-FLAG-2xStrepII)-ORF2(14xHis-UBQLN1), along with pET28a-6xHis-MBP-3C protease. Expression was induced with 0.4 mM IPTG at 16 °C for 20 h. Cells from a 20 ml culture were lysed in buffer A (50 mM Tris–HCl, pH 7.5, 200 mM NaCl, 5% glycerol, 1 mM β-mercaptoethanol, and 1 mM PMSF) by sonication on ice. The lysates were centrifugation at 20,000*g* for 30 min to separate the supernatant (S) and pellet (P).

To purify the UBQLN1, ATP5G1, and RNF126 complex from *E. coli*, cells were cotransformed with either pDuet-ORF2(14xHis-UBQLN1) or pDuet-ORF1(MBP-3C-ATP5G1-FLAG-2xStrepII)-ORF2(14xHis-UBQLN1), along with pET28a-6xHis-RNF126. Cells from a 200 ml culture were harvested and lysed as described previously. The clarified supernatant was incubated with Dextrin beads at 4 °C for 2 h. The resin was washed three times and eluted with 10 mM D-Maltose in lysis buffer. The elution was then incubated with Strep-Tactin beads at 4 °C for 90 min. The resin was washed twice and eluted with saturated biotin in lysis buffer (pH adjusted to 7.4).

### *In vitro* ubiquitination

Ubiquitination of rUBQLN-pATP5G1 or rUBQLN1-Omp25 was performed in a 100 μl reaction. Briefly, about 60 ng of ATP5G1–UBQLN1 complexes were used along with purified proteins including 6xHis-TEV-ubiquitin (or 2xStrepII-ubiquitin) (15 μM), 6xHis-Ube1 (0.5 μM), UbcH5b (0.5 μM), 6xHis-RNF126 (2.5 μM), and 2 mM ATP (Roche; catalog no.: 10519979001). All ubiquitination reactions were carried out in buffer containing 30 mM Hepes, pH 7.4, 50 mM NaCl, 2 mM MgCl_2_, and 0.25 mM DTT at 25°C. Ubiquitination reactions were terminated at different time points by SDS sample buffer. Samples were either directly analyzed by immunoblotting or diluted into 1 ml IP buffer (30 mM Tris–HCl, 150 mM NaCl, and 1% TX-100). The supernatants were incubated with Strep-Tactin beads at 4 °C for 2 h. Beads were then washed three times with 1 ml IP buffer, eluted with 50 μl SDS sample buffer, and analyzed by immunoblotting.

### CHX experiment

For CHX (Sigma–Aldrich; catalog no.: C7698) chase experiments, ATP5G1-3xFLAG stable-expressing cells grown to ∼90% confluency in 12-well plates were first pretreated with 4 μM CCCP and 2 μM valinomycin for 60 min. After pretreatment, the cells were further treated with either 10 μM MLN7243 or 10 μM MG132 for 15 min. Subsequently, the cells were added with 100 μg/ml CHX for various times. The cells were then lysed with SDS sample buffer for SDS-PAGE and immunoblot analysis.

### Protein degradation in cytosol fraction

HEK293T cells cultured in 15 cm dish (∼90% confluency) were washed once with 1× ice-cold PBS and collected with 1 ml PBS. After brief centrifugation (500g, 3 min), the cell pellet was resuspended in 0.5 ml buffer (25 mM Hepes, pH 7.4, 10% glycerol, 10 mM MgCl_2_, and 1 mM DTT), and lysed by ∼20 passages through a 25-gauge needle using a syringe of 2 ml. The lysate was centrifuged at 10,000*g* for 10 min to remove heavy membranes, followed by centrifugation at 100,000*g* (Beckman TLA100.3 rotor) for 60 min to remove membranes. The cytosol fractions were collected and filtered through a 0.45 μm filter. The cytosol fractions were treated with or without various inhibitors (*e.g.*, CB5083, MG132, MLN7243) for 15 min on ice. Next, 120 ng rUBQLN1–pATP5G1 binary complex was added to 200 μl of cytosol containing 1× energy regenerating system (20xERS: 20 mM ATP, 20 mM GTP [Roche; catalog no.: 10106399001], 0.8 mg/ml creatine kinase [Roche; catalog no.: 127566], and 200 mM creatine phosphate [Roche; catalog no.: 621714]). The mixture was incubated at 37°C, and the reaction was stopped at different time points by adding SDS sample buffer before immunoblot analysis.

## Data availability

Original scans of the gels and blots are available upon request.

## Conflict of interest

The authors declare that they have no conflicts of interest with the contents of this article.
